# Group comparisons of the individual electroretinogram time trajectories for the ascending limb of the b-wave using a raw and registered time series

**DOI:** 10.1186/s13104-023-06535-4

**Published:** 2023-09-29

**Authors:** Marek Brabec, Paul A. Constable, Dorothy A. Thompson, Fernando Marmolejo-Ramos

**Affiliations:** 1 Institute of Computer Science of the Czech Academy of Sciences, Prague, Czech Republic; 2 Flinders University, College of Nursing and Health Sciences, Caring Futures Institute, Adelaide, SA Australia; 3 The Tony Kriss Visual Electrophysiology Unit, Clinical and Academic, Department of Ophthalmology, Great Ormond Street Hospital for Children NHS Trust, London, UK; 4 UCL Great Ormond Street Institute of Child Health, University College London, London, UK; 5 Centre for Change and Complexity in Learning, The University of South Australia, Adelaide, Australia

**Keywords:** Electroretinogram, Time domain analysis, Curve registration, b-Wave

## Abstract

**Objectives:**

The electroretinogram is a clinical test commonly used in the diagnosis of retinal disorders with the peak time and amplitude of the a- and b-waves used as the main indicators of retinal function. However, subtle changes that affect the shape of the electroretinogram waveform may occur in the early stages of disease or in conditions that have a neurodevelopmental or neurodegenerative origin. In such cases, we introduce a statistical approach to mathematically model the shape of the electroretinogram waveform that may aid clinicians and researchers using the electroretinogram or other biological signal recordings to identify morphological features in the waveforms that may not be captured by the time or time–frequency domains of the waveforms. We present a statistical graphics-based analysis of the ascending limb of the b-wave (AL-b) of the electroretinogram in children with and without a diagnosis of autism spectrum disorder (ASD) with a narrative explanation of the statistical approach to illustrate how different features of the waveform based on location and scale derived from raw and registered time series can reveal subtle differences between the groups.

**Results:**

Analysis of the raw time trajectories confirmed findings of previous studies with a reduced and delayed b-wave amplitude in ASD. However, when the individual time trajectories were registered then group differences were visible in the mean amplitude at registered time ~ 0.6 suggesting a novel method to differentiate groups using registration of the ERG waveform.

**Supplementary Information:**

The online version contains supplementary material available at 10.1186/s13104-023-06535-4.

## Introduction

The electroretinogram (ERG) waveform is the summed electrical response of the retina to a brief flash of light over time. It varies in shape depending on the state of retinal adaption and the duration, color, strength, and frequency of the light flash [1]. The clinical ERG is used routinely to aid the diagnosis of retinal diseases that may be acquired [2] or inherited [3]. The ERG waveform has a negative trough termed the a-wave followed by a positive peak termed the b-wave that derive largely from the photoreceptors and bipolar cells of the retina respectively [1, 4]. On the ascending limb of the b-wave (here termed the AL-b) small peaks are observed termed the Oscillatory Potentials (OPs) whose origins derive from amacrine cells and contribute to the overall shape of the b-wave [5].

Several strategies have been applied to the analysis of the ERG waveform to reveal the underlying physiological process and how they may change with disease state. The a-wave has been modelled to study the kinetics of the phototransduction cascade that shapes the rate at which the first negative trough develops [6–8]. The OPs have been more difficult to define with different approaches applied including the summated amplitudes of the peaks and their corresponding peak times or the integrated root-mean-square amplitude of the OPs [4], or by applying wavelet analysis as a continuous [9] or discrete transform [10]. The development and application of the discrete wavelet transform to ERG signal analysis has provided an additional method to explore the time–frequency domain of the ERG and relate these parameters to the main retinal (ON and OFF) signalling pathways and the OPs [11–13] in retinal [14, 15] and neurodevelopmental disorders [16]. More recently, variable frequency complex demodulation has also been applied as an additional method to identify features for group classification [17]. Modelling of the b-wave amplitude luminance response function under dark [18] and light adapted [19] conditions has also been employed to assess retinal function. Specifically in the case of the light-adapted luminance response series to evaluate the relative contributions of the ON and OFF pathways to the photopic hill [19–21]. Whilst these methods are all appropriate, we introduce an additional approach based on point-by-point examination of measures of location and scale of the real and registered time series of the AL-b in control and ASD participants.

This report provides a description of another analytical approach to the ERG waveform that may be helpful in studies where differences in the shape of the waveform may augment standard measures of amplitude and time of the principal minima (a-wave) and maxima (b-wave) of the ERG waveform. Our main intention here is to illustrate this methodology using results previously reported in autism spectrum disorder (ASD) and control children with traditional measures of ERG analysis [21, 22]. In this case we used a sample of these groups ERG waveforms to demonstrate that by using raw and registered time series of the AL-b it was possible to identify characteristics of the shape that were not characterized by the a- and b-waves peak time and amplitude.

## Methods

We explored the details of *individual* time trajectories of the ERG recordings, focusing on the interval AL-b. Technically speaking, we viewed individual ERG curves as functional datapoints (or “functional observations”) in the sense of functional data analysis (FDA) [23–25]. To this end we interpolated the discrete time observations in a piecewise linear way (or using polygonal basis in the language used in the book by Ramsay et al. [26]. We were interested in the mean trajectory of the ERG signal (computed time-pointwise across individuals), and other features such as inter-individual variability expressed as time-pointwise standard deviations computed across individuals. Since we suspected the presence of outliers at least for some time points in the AL-b interval of interest, we compared the mean and standard deviation curves (as standard estimates of location and scale) to those based on more robust and outlier-resistant estimates (namely median and the mean absolute deviation (MAD) [27]. Next, we recalled from the standard notions of FDA and its applications [23, 25] that the ERG mean, and standard deviation trajectory (or more generally, location and scale curves) might not tell the whole story. Namely, different individual ERG waveforms might have common features such as local minima or local maxima, but their timing could easily be inter-individually variable. We wanted to separate-out these inter-individual variations in the timings of the a-wave minima and b-wave maxima and observe the overall shape of the AL-b interval. To this end, we normalized the curves through registration, so that each of them was scaled into [0,1] intervals in both the vertical (amplitude) and horizontal (time) axes (the a-wave minima time point then corresponded to time = 0, and the time point of the b-wave maxima corresponded to time = 1. This was a simplified version of the so-called ‘curve registration’ described by Ramsay and Silverman [25].

### Data set

The original ERG waveforms were recorded as part of a luminance response series in case (with) and control (without) an ASD diagnosis. The methods have been reported in detail previously [16, 21, 22] with all recordings made with the RETeval (LKC Technologies Inc., Gaithersburg, MD, United States) with skin electrodes (right eye always first). ERGs were recorded using a custom Troland protocol (undilated pupils) with white flashes presented at 2 Hz with equivalent strength of 1.2 log cd s m^−2^ on a 40 cd m^−2^ white background filtered between 0.3–300 Hz with 30–60 averages used to generate the reported waveform. There were 52 ERG waveforms (29 from the right eye) from 26 control participants and 51 waveforms (34 from the right eye) from 24 ASD individuals included in the dataset with the following demographics for each group: (Mean ± SD): Age (ASD 12.46 ± 2.66, control 11.84 ± 2.75), and female sex ASD 23.5% and 50% control. ASD participants were recruited from existing databases or local autism groups and via social media at the study sited based in London or Adelaide. The ASD participants met diagnostic criteria based on the Diagnostic Statistical manual (DSM) IV-TR [28, 29] or V [30] with clinical evaluations performed by experienced child psychologists or psychiatrists using a combination of observation and clinical tools (ADOS-2 and 3di) [31–33]. Control children were recruited from social media and word of mouth and had no developmental delay with normal visual function and no history of ocular surgery.

This study was approved by the Flinders University Human Research Ethics Committee and the Southeast Scotland Research Ethics Committee in the United Kingdom and conformed to the tenets of the declaration of Helsinki. Written informed consent to participate in this study for those under 16 years of age was provided by the participants’ legal guardian/next of kin with permission to re-use any data for future studies.

## Results

**Fig. 1:**
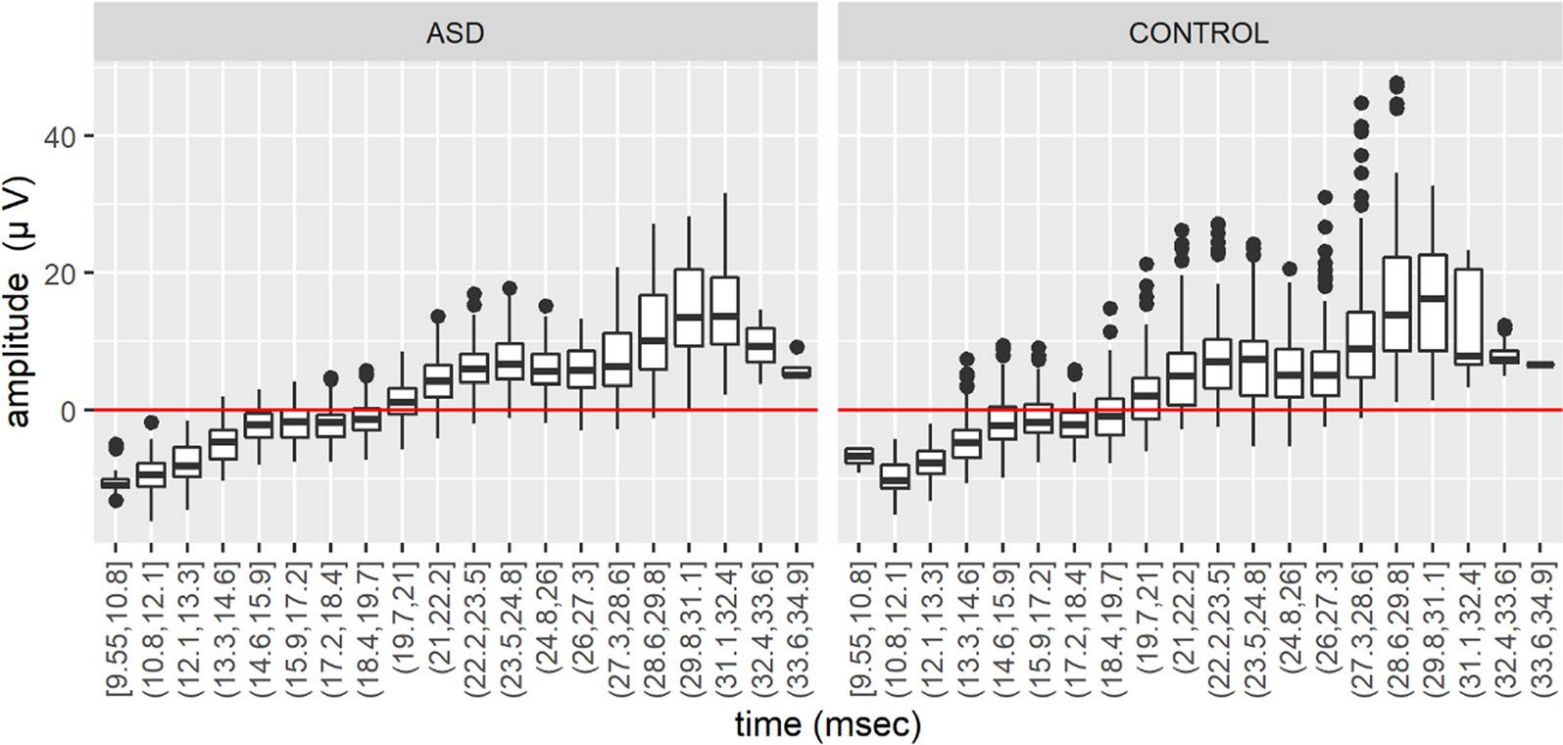
Raw boxplots incorporating the ascending limb of the b-wave (AL-b) interval for autism spectrum disorder (ASD) and control groups based on time-pointwise intervals from 9.55 to 34.9 ms. The ASD group have less variability in the range of values for the amplitude compared to controls with fewer outliers

Figure 1 displays the raw data of the boxplots representing the interquartile range with the median as a measure of location and the whiskers as a measure of scale for the AL-b trajectories in the time-pointwise intervals. Outliers are shown as filled circles which are more numerous in the control group indicating greater variability than the ASD group. For additional detail on the ERG waveform and see Additional file 1.

### Individual trajectories

**Fig. 2:**
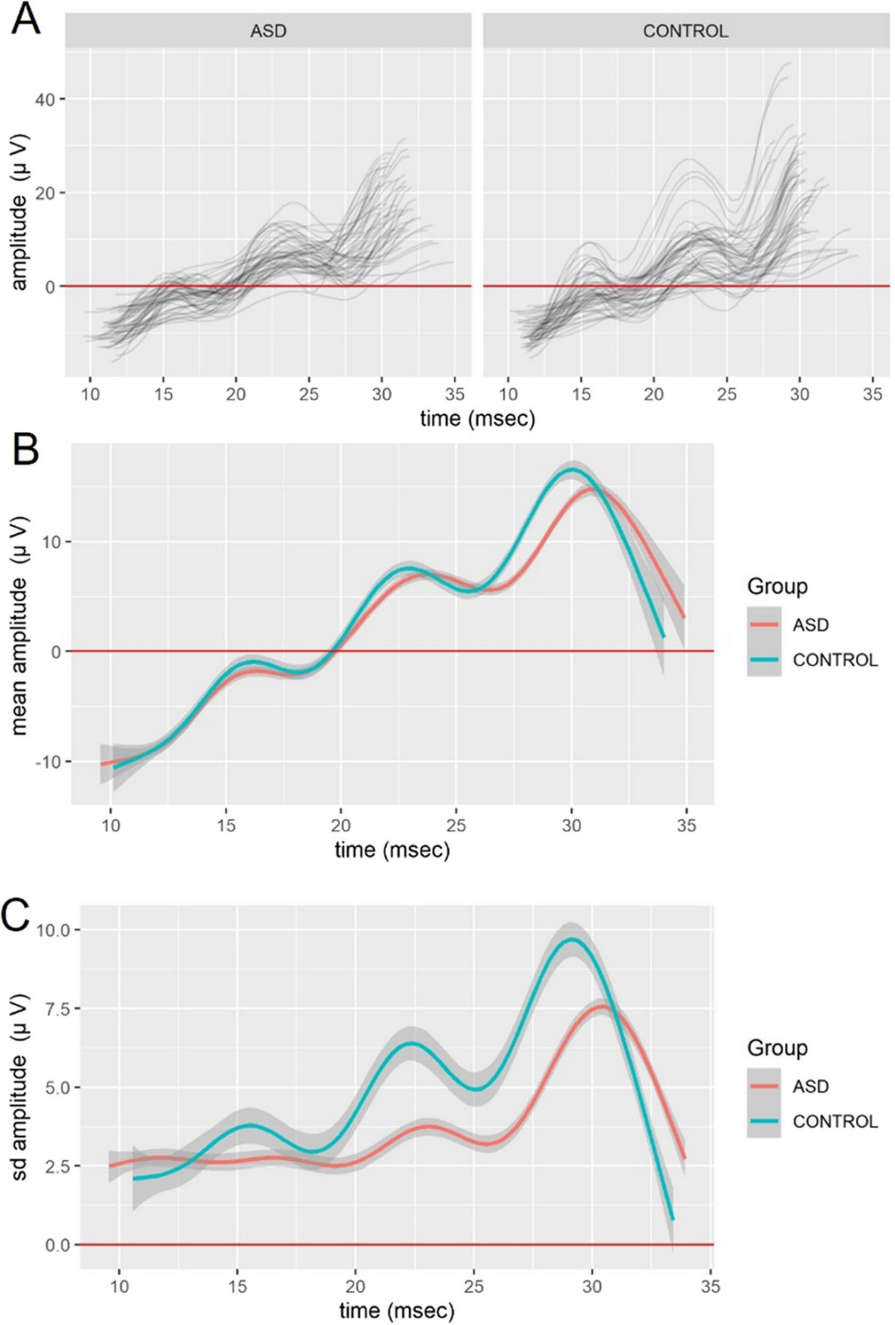
**a** Raw data of the individual trajectories of the electroretinogram (ERG) waveforms in the time interval of the ascending limb of the b-wave (AL-b) for autism spectrum disorder (ASD) and control participants at flash strength of 1.2 log cd s m^−2^. The control group showed a higher maximum value for the b-wave and greater variability in the amplitudes of the second peak of the oscillatory potentials (OPs) visible in the time interval of ~ 20–25 ms compared to the ASD group. **b** Plots of the mean trajectory of the AL-b for the groups illustrating the overall differences in the general shape of the AL-b. The control group has an overall higher amplitude occurring at approximately 30 ms with more prominent OPs on the AL-b visible as larger peaks. **c** The standard deviation of the raw AL-b interval. There is a greater variation in the amplitudes observed in the control group for the first and second peak compared to ASD. Of note is that the ASD group’s peaks show a slight delay as well as less variability in amplitude comparted to the controls. Shaded areas represent 95% pointwise-constructed Confidence Intervals

When the raw signals from the ERG waveforms were plotted incorporating the AL-b interval at 1.2 log cd s m^−2^ the overall ‘shape’ of the waveforms were similar with two small peaks from the OPs apparent before the b-wave maxima. The main observations from the raw ERG trajectories in the AL-b interval between the groups was that the control reached a higher b-wave maximum and that the two smaller OPs peaks on the AL-b were visible for both groups although the second peak was more pronounced and more variable for the control group (see Fig. 2a).

### Mean trajectories of the raw waveforms

Figure 2b shows the mean of the individual trajectories for the raw ERG waveforms for the two groups incorporating the AL-b time interval accompanied with the pointwise-constructed 95% confidence intervals. Not only that the confidence intervals describe uncertainty in the mean estimates, but the overlap/non-overlap of the group-specific confidence intervals at a particular time (overlap/non-overlap of the grey regions at a particular vertical section) can be used to obtain pointwise tests of difference among the mean trajectories. If the grey areas do overlap, the test of null hypothesis of both groups having the same mean is not significant at 5% level. If they do not overlap, the test is significant at 5% level. By scanning for non-overlap regions of time, we can easily see where the mean trajectories are significantly different between the groups (same approach can be used for other characteristics in subsequent figures). Inspection of the mean trajectories within the AL-b showed a reduced amplitude and delayed time to peak of the b-wave maxima for the ASD group which has been previously reported for this flash strength [21]. The other notable difference was the mean trajectories of the OPs had a slightly higher amplitude and earlier peak in the control group compared to the ASD group. These observations in the different nature of the OPs in ASD adults have been noted before with a notch appearing in OP2 in adults with ASD [20] and in the ERG spectra of the OPs in ASD the energy is lower compared to controls in the higher OPs frequency components [16].

When the median rather than the mean of the AL-b interval was plotted a similar profile was evident-indicating that the mean plot was not contaminated by outliers (see Additional file 1: Figure S2). Overall, the mean and median plots (as two estimates of statistical location) of the AL-b interval were similar and showed that the waveforms followed a similar trajectory with greater divergence towards the b-wave peak which are consistent with previous studies in this population group [16, 20–22].

### Standard deviation trajectories of the raw waveforms

Figure 2c shows the standard deviation containing the region of the AL-b interval for the ASD and control groups which displays similar characteristics to the MAD as another statistical estimate of scale (see Additional file 1: Figure S3). Here the standard deviation of the ERG waveform illustrates the greater overall variance in the control group with a larger range of amplitudes centered around ~ 16 and 22 ms on the AL-b corresponding to the OPs peaks.

### Mean trajectories of the registered waveforms

**Fig. 3:**
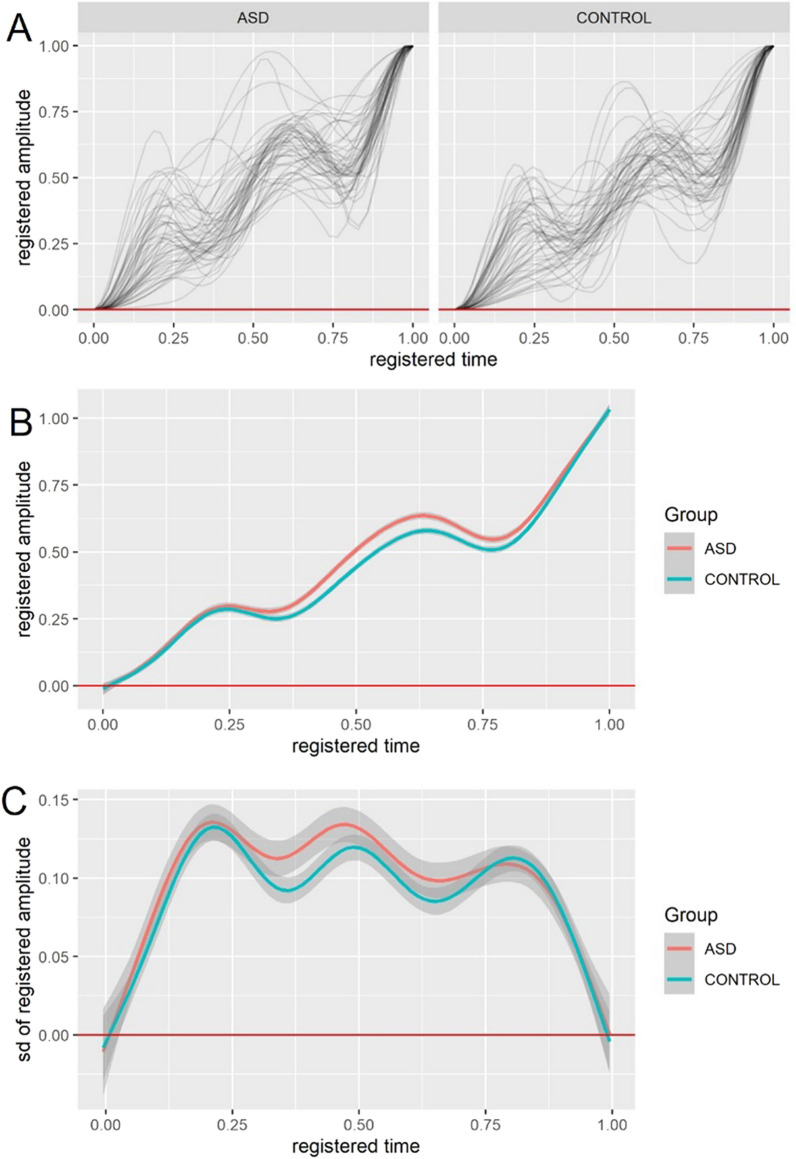
**a** Registered waveforms for the ascending limb of the b-wave (AL-b) individual trajectories for the autism spectrum disorder (ASD) and control groups. For each group t = 0 represents the a-wave minima and t = 1 the time of the b-wave maxima so that all data are normalized to a unitary scale in time and amplitude. **b** Mean registered AL-b intervals for the groups showing that at registered time ~ 0.6 there is a group difference in the mean registered amplitude between the ASD and controls. **c** Standard deviation of the registered individual waveforms for the AL-b with both groups showing a strong overlap following registration of this interval. Shaded areas represent the 95% pointwise-constructed Confidence Intervals

To obtain the registered plots, the raw amplitude and time series of the AL-b intervals were registered so that the point [0,0] corresponded to the a-wave minima and the point [1,1] corresponded to the b-wave maxima (Fig. 3a). This registration or normalizing step allowed a different insight into the AL-b signal trajectories. By fixing the scale of time and amplitude to 1.0 so that the general shape of the AL-b interval could be observed independently of the raw time scale. Registration of the signal reduced the influence of the phase and amplitude variability between the groups by normalizing theses axes to a unitary scale- enabling a direct comparison of the AL-b shape that was independent of the real time scale (see Fig. 3a).

Figure 3b shows how the mean registered signal for the ASD group was on average greater at ~ 0.6 of the registered time indicating that when the AL-b segment was normalized then at 0.6 of the raw time intervals between the a-wave minima and the b-wave amplitude the signal amplitude was higher in the ASD group compared to the control group.

### Standard deviation trajectories of the registered waveforms

Figure 3c shows the standard deviation of the registered data which has greater overlap between the groups suggesting less variability in the statistical scale measures of the registered data between these groups compared with the raw AL-b interval. This difference between the raw and registered standard deviation may be due to the registration of the AL-b interval to a unit scale resulting in less variability between groups associated with differences in the real time point at which the amplitudes changed. Note the standard deviation scale in the registered plot (Fig. 3c) is a factor 10 less than the raw values of the standard deviation shown in Fig. 2c. See Additional file 1: Figures S4–S6 for plots of registered data showing box plots, median and MAD respectively.

## Discussion

This introduction to the analysis of the ERG waveform based some of the principles of FDA, using signal registration [24, 25], may provide an additional methodological approach to the analysis of the ERG waveform. Here, we limited the analysis to one region of interest, the AL-b, whose shape is influenced by bipolar, amacrine and glial cells whose timing of depolarizing and hyperpolarizing currents and the state of retinal adaption will affect the overall shape of the AL-b interval [5, 34–37]. In this instance, the intention was not to provide a detailed group comparison of mean values obtained using this method, but to present a different representation of ERG data that could assist in future studies using the ERG where traditional measures of the median values of the amplitude and time to peak of the main a- and b-waves are used to identify group differences [1, 4].

In this example, by comparing the differences between ASD and control raw and registered ERG waveforms with estimates of location and scale for the AL-b interval differences in the overall shape and variability between the groups could be related. The raw data plots were consistent with previous observations of a reduced and delayed b-wave amplitude in ASD for this flash strength [21, 22], whilst the registered plots revealed a larger mean amplitude at registered time of ~ 0.6 in the ASD group that was not noticeable in the raw data (see Fig. 1b). Thus, registration may reveal earlier and more subtle differences in the shape of specific intervals within the ERG, that may not be revealed when examining solely the amplitude and time to peaks of the principal components as is routinely reported for the clinical ERG [1, 4].

The registration of the AL-b interval between the a-wave minima and b-wave maxima to a fixed unitary scale, enabled the proportional differences between measures of location to be visualized. The main difference observed through this analysis was that the mean ERG registered amplitudes were provided a clearer separation of the groups compared to the raw AL-b trajectories that were location based. This observation highlighted that through registration, additional group differences may be revealed that may not be apparent in the raw signal. In this example rescaling of the time interval for each group reduced the influence of phase variations in the AL-b raw time interval revealing the higher registered signal value at about 0.6 for the ASD group.

From these observations of location and scale one could infer that the AL-b differs between the groups with less contribution of the later OPs to the overall amplitude of the AL-b in ASD that has been previously observed using spectral analyses of the ERG [16, 17]. The inference being that the contribution of the amacrine cells to the timing and amplitude of the OPs differs in ASD compared to controls [5]. These observational difference between the registered and raw trajectories between groups could also be due to differences in the generation of the light-adapted a-wave whose origins stem from a mixture of mainly cone hyperpolarization with some second order neural inputs as well [35, 37]. These multiple factors would influence the a-wave minima and subsequent shape of the registered AL-b interval because the origin at [0,0] will depend upon the real raw time a-wave minima. These group differences from the registered AL-b interval at time ~ 0.6 were not apparent through conventional time or spectral domain analysis of the whole ERG waveform reported previously [16, 20–22] and may offer an additional classification marker in this representative example of the application of FDA principles to ERG waveform analysis.

The choice of mean or median or MAD vs SD as estimates of location and scale is arbitrary as the general findings between the groups is qualitatively similar in this case. The strength of FDA is to allow a point-by-point analysis of the waveform’s physiological shape based on its location (mean or median) and the inter-individual variability based on measures of SD or MAD. The interquartile range of the SD is larger but either measure will provide insights to the specific time points in which group differences are significantly different. Thus, this method may give additional information regarding localized changes within the overall waveform’s shape that may not be captured by measures of amplitude and peak time. Registration of the waveform provides a method in which to quantitatively observe differences in location and scale between groups on the same time scale and would enable a more formal analysis of the variations in the waveform. This may help to highlight specific parts of the waveform that are different such as the shape of the OPs or turning points of the a- and b-wave or the rate of change in the amplitude between groups on a unified time scale. The statistical values of location or scale at real time or registered time points could then be used in statistical learning models as features with which to classify either groups [17] or potentially phenotypic differences in retinal diseases [38]. Features of the generated curves that differentiate groups could be use din decision tress to further support group classification [39].

This initial study therefore supports the potential of FDA analysis of the AL-b interval of the ERG using estimates of location and scale that may be affected in retinal disease [3, 40]. Further time-trajectory analyses using raw and registered intervals containing the descending limb and turning point of the a-wave, relating to phototransduction [8, 35, 37] or the shaping of the descending limb of the b-wave and the PhNR by and ganglion cells [41] may contribute further to disease diagnosis and classification [3, 40, 42–45]. To identify the optimal time interval in which to examine group differences, the region where there is greatest separation of the confidence interval boundaries could be used to localize the most suitable time or registered time point or interval. Thus, this report introduces the potential analysis of the ERG waveform using elements of FDA theory as an additional tool with which to analyze the ERG in retinal and neurological disorders [17, 46–48].

FDA has been implemented to aid diagnosis in cardiology using electrocardiogram signals [49], gait patterns in Parkinson’s disease [50] and electroencephalograph recordings to assess driver fatigue [51]. Here our focus is on the ERG, and this method may therefore assist in expanding the application of the ERG to not only retinal [46] but also in the classification neurological disorders where multiple genetic and environmental factors may interact to hinder the identification of distinct phenotypical entities based on conventional ERG measures [38, 48, 49, 52, 53].

## Limitations

In this preliminary report we limited the analysis to a statistical graphics-based analysis of the waveforms as drawing inferences about statistical group differences have been previously reported and the intention was not to report on the physiological meaning of the findings with respect to ASD and controls. In future we will report these findings including subjects from related neurodevelopmental conditions such as attention deficit hyperactivity disorder (ADHD) and in cases where subjects meet more than one diagnostic criterion such as ASD and ADHD. Further limitations on the analysis are that we did not inspect other regions of the ERG waveform such as the turning points of the a- and b-waves or the descending limb of the b-wave that is referred to as the photopic negative response. These features would be important in other studies of the ERG where the hyperpolarization of the photoreceptors and contribution of the ganglion cells are important. In addition, we focused here on measures of location and scale but not shape such as kurtosis which may also provide a valuable perspective on the group differences.

### Supplementary Information

**Additional file 1:** Contains raw and additional plots of location and scale.

## Data Availability

Raw waveform data and Rcode used in this analysis is available at: https://doi.org/10.25451/flinders.21546210.v1
