# Supplementary material for: Group comparisons of the individual electroretinogram time trajectories for the ascending limb of the b-wave using a raw and registered time series
Source: BMC Res Notes. 2023 Sep 29;16:238. doi: 10.1186/s13104-023-06535-4 (PMC10542250; doi:10.1186/s13104-023-06535-4)
Supplement: Supplementary file 1 — Additional file 1: Contains raw and additional plots of location and scale. [file 13104_2023_6535_MOESM1_ESM.pdf]

Additional Material

Group comparisons of the individual electroretinogram time trajectories for the ascending limb of the b-wave using a raw and registered time series

Marek Brabec, Paul A. Constable, Dorothy A. Thompson & Fernando Marmolejo-Ramos

Table of Contents

The Electroretinogram 2
Raw data 3
Raw Group plots of AL-b for scale and location 3
Registered Data 4
Registered Group plots of AL-b for scale and location 5
References 7

The Electroretinogram

The clinically recorded electroretinogram (ERG) waveform results from the summation of activity of several neural generators within the retina. The dark-adapted ERG was first analyzed by Granit who defined three 'phases' PIII, PII and PI which were labelled according to their resistance to ether anesthesia [1] and related to the initial negative 'a-wave', the following positive 'b-wave' and a later smaller positive 'c-wave', respectively. The light adapted ERG is also the product of the summed retinal generators that give rise to the characteristic negative a-wave followed by the positive b-wave. A typical light-adapted ERG waveform is shown in Additional Figure S1 with the initial a-wave trough that derives from the hyperpolarization of the photoreceptors [2] with some contribution from second order neurons [3, 4]. The second peak, termed the b-wave is formed by the depolarization of the bipolar cells [5, 6], glial cell potassium currents [7] and spiking amacrine cells initiating the oscillatory potentials (OPs) that appear as small wavelets on the Ascending Limb of the b-wave [8-12]. The descending limb of the b-wave is further shaped by contributions of the ganglion cells [13] and forms what is termed the photopic negative response (PhNR) whose amplitude can be measured from either the peak of the b-wave or from the baseline to a selected minimum point following the b-wave peak [14]. The interpretation of quantitative measures of amplitudes and peak times, together with a qualitative appraisal of waveform shape are then used in the clinical application of the ERG [15].

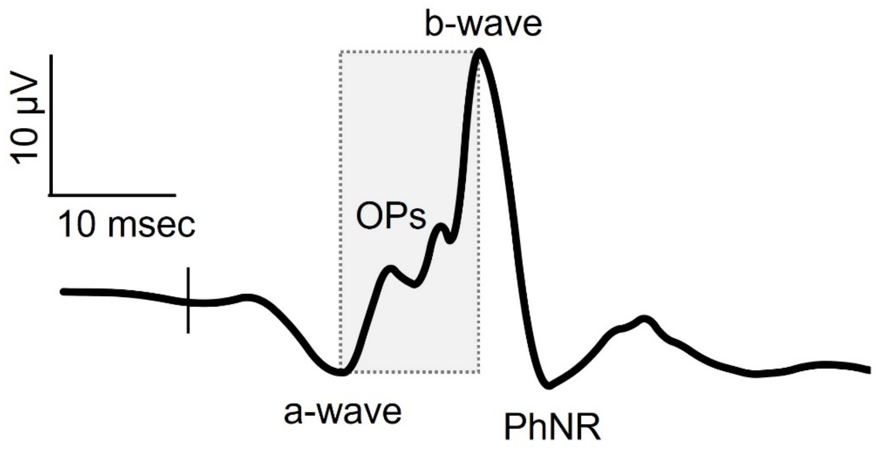

Additional Figure S1. The light adapted electroretinogram (ERG) waveform from a typical individual shows the initial negative a-wave (mainly derived from cone photoreceptors), the positive peak of the b-wave (bipolar and glial cells) with the contribution of the amacrine cells generating the oscillatory potentials (OPs) that appear as ripples on the ascending limb of the b-wave (AL-b) shaded area. The descending limb of the b-wave is shaped by ganglion cells that form the Photopic Negative Response (PhNR) that typically reaches a minimum at approximately 72 ms after the brief light flash stimulus represented by the vertical line preceding the a-wave.

Several strategies have been applied to the analysis of the ERG waveform to reveal the underlying physiological process and how they may change with disease state. The a-wave's kinetics have been modeled to study the phototransduction cascade that shapes the rate at which the first negative trough develops [16-18]. The OPs have been more difficult to define with different approaches applied consisting of the summated amplitudes of the peaks

and their corresponding peak times or the integrated root-mean-square amplitude of the OPs [15], or by applying wavelet analysis as a continuous [19] or discrete transform [20]. The development and application of the discrete wavelet transform to ERG signal analysis has provided an additional method to explore the time-frequency domain of the ERG and relate these parameters to the main retinal (ON and OFF) signaling pathways and the OPs [21-23] in retinal [24, 25] and neurodevelopmental disorders [26]. In addition, mathematical modelling using a stochastic approach has also been applied based on the P1, PII and PII components of the ERG [27]. Modelling of the b-wave amplitude luminance response function under dark [28] and light adapted [29] conditions has also been employed to assess retinal function. Specifically in the case of the light-adapted luminance response series to evaluate the relative contributions of the ON and OFF pathways to the photopic hill [29-31].

Raw data

Raw Group plots of AL-b for scale and location

Additional Figure S2 shows the median plot containing the AL-b (ascending limb of the b-wave) interval for the control and ASD groups raw ERG waveforms. The median plot is akin to the plot of mean signal amplitude, the control group showed an earlier and larger b-wave peak occurring at approximately 28 msec with the second OPs peak also occurring earlier. The mean or median plots give a similar indication of location for the two groups.

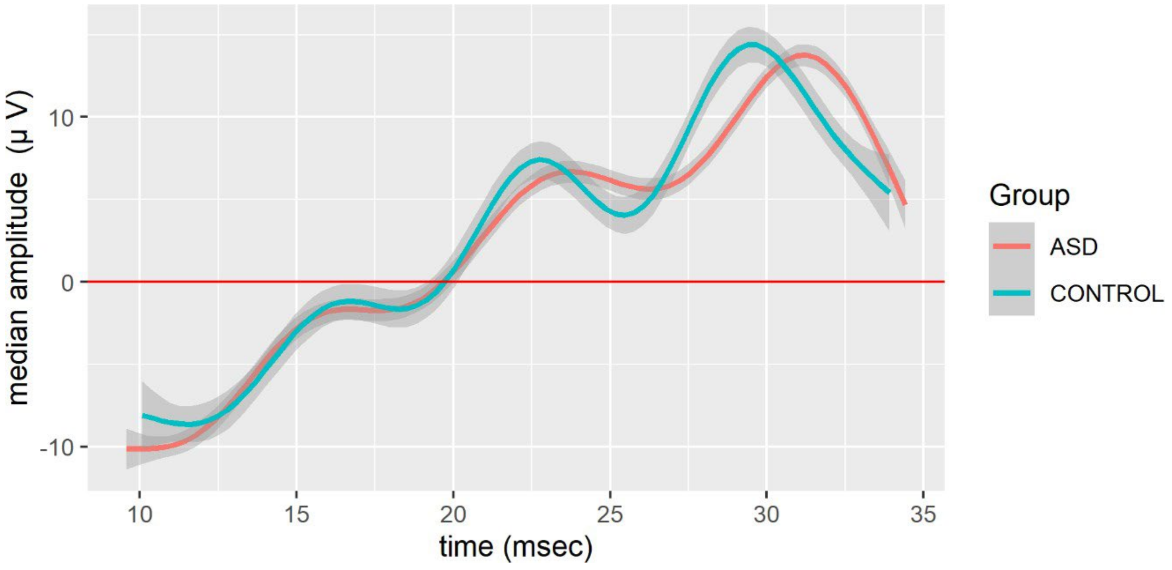

Additional Figure S2. Raw waveform of the median values for the electroretinogram waveform for ASD and control groups at 1.2 log phot cd.s.m^{-2} incorporating the AL-b region. The control group have a larger response with an earlier second peak and b-wave amplitude than the ASD group. Shaded area represents 95%CI.

Additional Figure S3 shows the mean absolute deviation (MAD) as a measure of scale for the raw

ERG waveforms between ASD and control. The MAD follows a similar profile to the standard deviation illustrated in the main paper indicating that both measures are compatible and not overtly influenced by outliers.

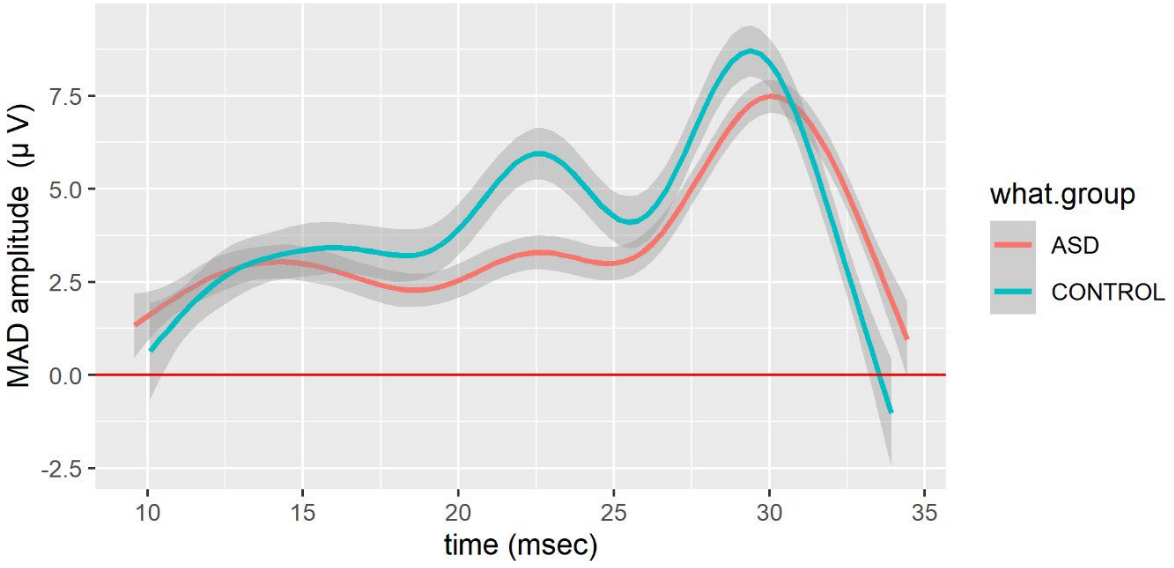

Additional Figure S3 The Mean Absolute Deviation for the raw signal for ASD and control groups. Consistent with the standard deviation plot the MAD also highlights a greater variability in the signal amplitude in the interval AL-b which relates the oscillatory potential contribution to this time interval. Shaded area represents 95%CI.

Registered Data

Additional Figure S4 shows the mean and interquartile range of the registered data with both groups raw data now normalized to a unitary scale on the time and amplitude axis. To note is that for some individuals in the ASD group the normalized signal value (amplitude) reaches ~1.0 at registered time ~0.6 which is not the case in the control group where all individuals reach a maximum signal value at registered time =1.0 The scale of the spread of data is larger in the initial 0-0.25 registered time interval suggesting greater variability in this region when the raw data is normalized to the unitary scale. Therefore, the registered plots give an alternative aspect to how the scale varies between groups when set to the same time scale between the a-wave minima and the b-wave maxima.

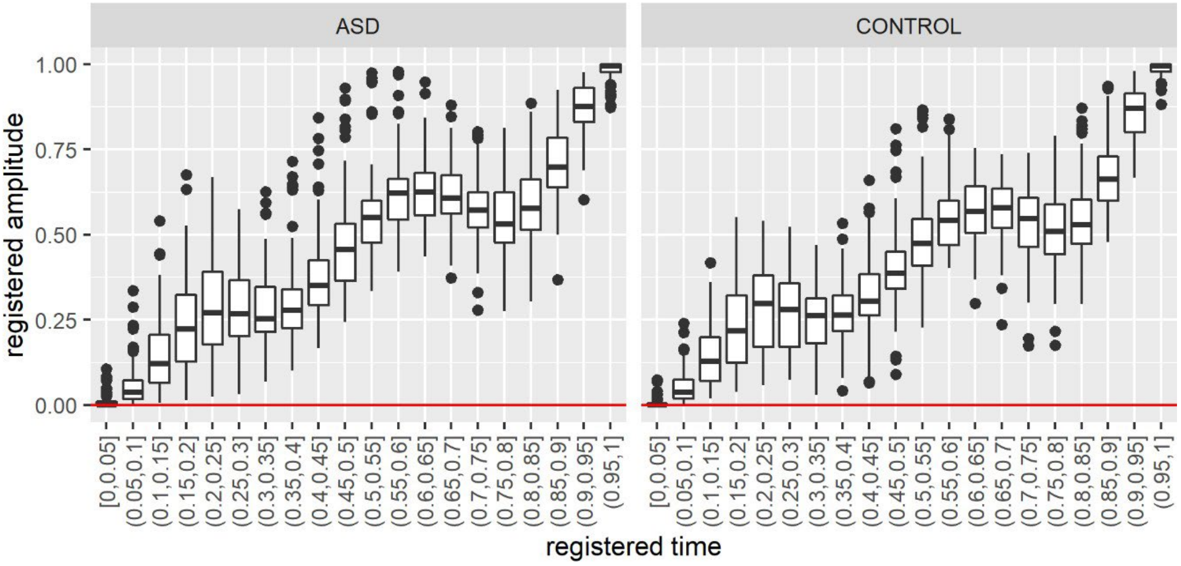

Additional Figure S4 Boxplots of the registered data shows how the AL-b of the ASD group in some instances is equivalent to the scale of the b-wave amplitude at about 0.6 registered time. In contrast at the similar registered time point the in control the amplitude is approximately 0.8 of the maxima. Plots show the mean with Interquartile range with outliers as filled circles within the time windows shown on the x-axis.

Registered Group plots of AL-b for scale and location

Additional Figure S5 shows the median plot of the registered signal values between ASD and control groups. The mean plot indicated a higher signal value for ASD at time =0.6 which is also apparent in the median plot. The normalizing of the time and amplitude to a unitary scale enables an alternative perspective on the shape of the AL-b independent of raw time and raw amplitude locations.

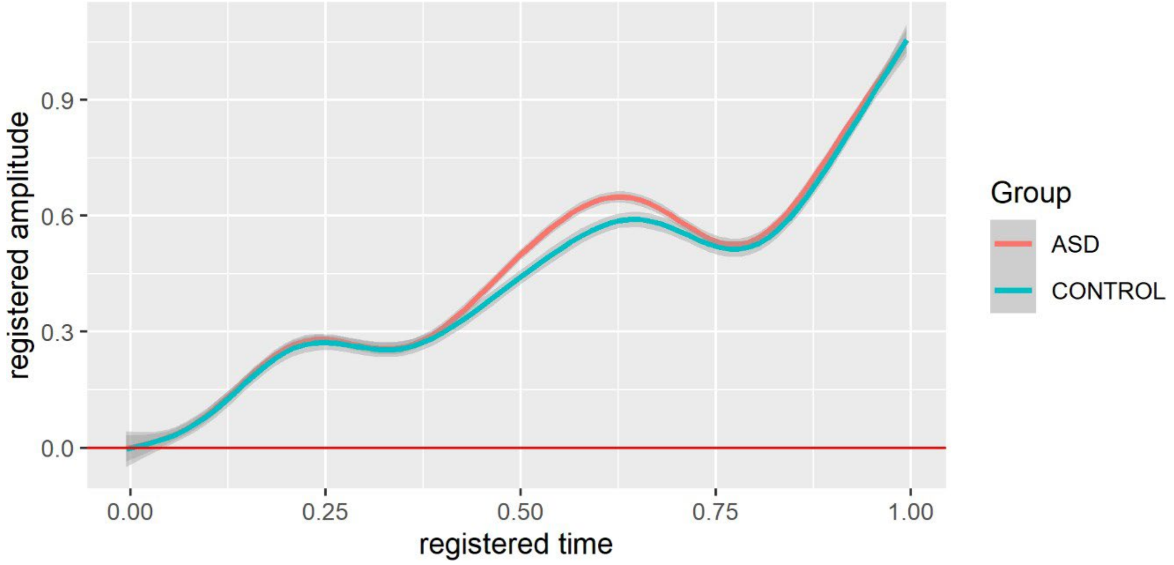

Additional Figure S5 The median signal for the registered data shows a similar difference between groups at registered time = 0.6. Shaded area represents 95%CI.

Additional Figure S6 as a measure of scale shows the MAD for the registered signal data with similar variability profile between groups. MAD is less resistant to outliers and hence this plot indicates less variability between groups than the plot of SD.

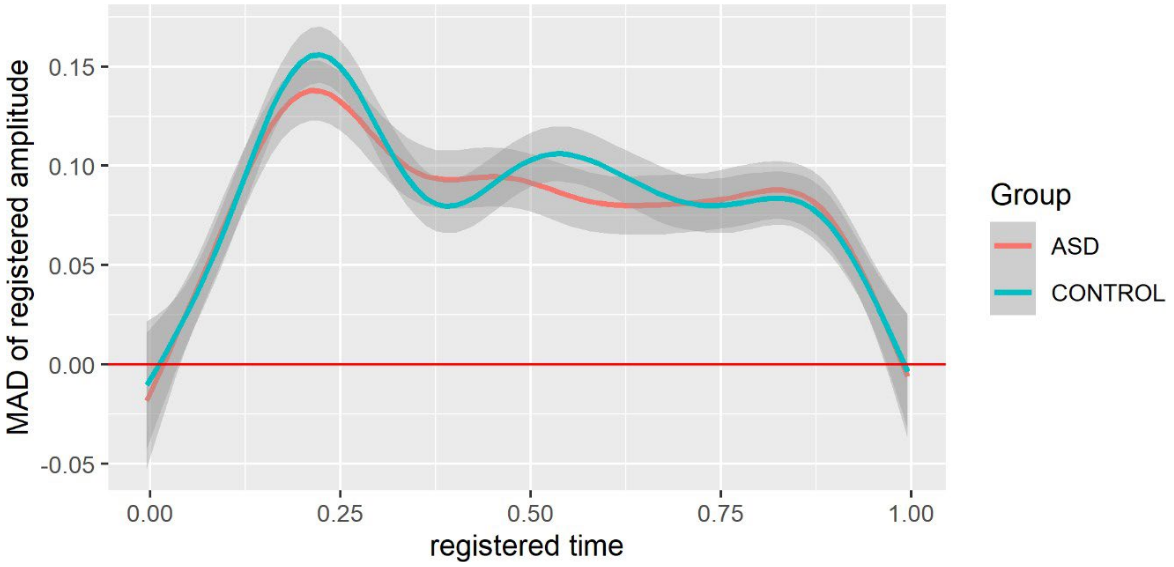

Additional Figure S6. Mean Absolute Deviation shows a similar profile for the registered data across both groups. Shaded area represents 95%CI.

References

1. Granit, R., *Physiology of vision.* Annu Rev Physiol, 1950. **12**: p. 485-502.
2. Baylor, D.A., T.D. Lamb, and K.W. Yau, *The membrane current of single rod outer segments.* J Physiol, 1979. **288**: p. 589-611.
3. Bush, R.A. and P.A. Sieving, *A proximal retinal component in the primate photopic ERG a-wave.* Investigative ophthalmology & visual science, 1994. **35**(2): p. 635-645.
4. Gouras, P. and C. MacKay, *A new component in the a-wave of the human cone electroretinogram.* Doc Ophthalmol, 2000. **101**(1): p. 19-24.
5. Heynen, H. and D. van Norren, *Origin of the electroretinogram in the intact macaque eye--II. Current source-density analysis.* Vision Res, 1985. **25**(5): p. 709-15.
6. Knapp, A.G. and P.H. Schiller, *The contribution of on-bipolar cells to the electroretinogram of rabbits and monkeys. A study using 2-amino-4-phosphonobutyrate (APB).* Vision Res, 1984. **24**(12): p. 1841-6.
7. Thompson, D.A., S. Feather, H.C. Stanescu, B. Freudenthal, A.A. Zdebik, R. Warth, et al., *Altered electroretinograms in patients with KCNJ10 mutations and EAST syndrome.* J Physiol, 2011. **589**(Pt 7): p. 1681-9.
8. Wachtmeister, L., *Some aspects of the oscillatory response of the retina.* Prog Brain Res, 2001. **131**: p. 465-74.
9. Wachtmeister, L., *Oscillatory potentials in the retina: what do they reveal.* Prog Retin Eye Res, 1998. **17**(4): p. 485-521.
10. Wachtmeister, L., *Further studies of the chemical sensitivity of the oscillatory potentials of the electroretinogram (ERG). II. Glutamate-aspartate-and dopamine antagonists.* Acta Ophthalmol (Copenh), 1981. **59**(2): p. 247-58.
11. Wachtmeister, L., *Further studies of the chemical sensitivity of the oscillatory potentials of the electroretinogram (ERG) I. GABA- and glycine antagonists.* Acta Ophthalmol (Copenh), 1980. **58**(5): p. 712-25.
12. Wachtmeister, L. and J.E. Dowling, *The oscillatory potentials of the mudpuppy retina.* Invest Ophthalmol Vis Sci, 1978. **17**(12): p. 1176-88.
13. Viswanathan, S., L.J. Frishman, J.G. Robson, and J.W. Walters, *The photopic negative response of the flash electroretinogram in primary open angle glaucoma.* Invest Ophthalmol Vis Sci, 2001. **42**(2): p. 514-22.
14. Frishman, L., M. Sustar, J. Kremers, J.J. McAnany, M. Sarossy, R. Tzekov, et al., *ISCEV extended protocol for the photopic negative response (PhNR) of the full-field electroretinogram.* Doc Ophthalmol, 2018. **136**(3): p. 207-211.
15. Robson, A.G., L.J. Frishman, J. Grigg, R. Hamilton, B.G. Jeffrey, M. Kondo, et al., *ISCEV Standard for full-field clinical electroretinography (2022 update).* Doc Ophthalmol, 2022. **144**(3): p. 165-177.
16. Mahroo, O.A., V.S. Ban, B.M. Bussmann, H.C. Copley, C.J. Hammond, and T.D. Lamb, *Modelling the initial phase of the human rod photoreceptor response to the onset of steady illumination.* Doc Ophthalmol, 2012. **124**(2): p. 125-31.
17. Friedburg, C., C.P. Allen, P.J. Mason, and T.D. Lamb, *Contribution of cone photoreceptors and post-receptoral mechanisms to the human photopic electroretinogram.* J Physiol, 2004. **556**(Pt 3): p. 819-34.
18. Smith, N.P. and T.D. Lamb, *The a-wave of the human electroretinogram recorded with a minimally invasive technique.* Vision Res, 1997. **37**(21): p. 2943-52.
19. Forte, J.D., B.V. Bui, and A.J. Vingrys, *Wavelet analysis reveals dynamics of rat oscillatory potentials.* Journal of Neuroscience Methods, 2008. **169**(1): p. 191-200.
20. Gauvin, M., A.L. Dorfman, N. Trang, M. Gauthier, J.M. Little, J.M. Lina, et al., *Assessing the Contribution of the Oscillatory Potentials to the Genesis of the Photopic ERG with the Discrete Wavelet Transform.* Biomed Res Int, 2016. **2016**: p. 2790194.
21. Gauvin, M., J.M. Lina, and P. Lachapelle, *Advance in ERG analysis: from peak time and

amplitude to frequency, power, and energy. Biomed Res Int, 2014. **2014**: p. 246096.

22. Gauvin, M., J.M. Little, J.M. Lina, and P. Lachapelle, *Functional decomposition of the human ERG based on the discrete wavelet transform.* J Vis, 2015. **15**(16): p. 14.
23. Gauvin, M., M. Sustar, J.M. Little, J. Brecelj, J.M. Lina, and P. Lachapelle, *Quantifying the ON and OFF Contributions to the Flash ERG with the Discrete Wavelet Transform.* Transl Vis Sci Technol, 2017. **6**(1): p. 3.
24. Dorfman, A.L., M. Gauvin, D. Vatcher, J.M. Little, R.C. Polomeno, and P. Lachapelle, *Ring analysis of multifocal oscillatory potentials (mfOPs) in cCSNB suggests near-normal ON-OFF pathways at the fovea only.* Doc Ophthalmol, 2020. **141**(2): p. 99-109.
25. Brandao, L.M., M. Monhart, A. Schötzau, A.A. Ledolter, and A.M. Palmowski-Wolfe, *Wavelet decomposition analysis in the two-flash multifocal ERG in early glaucoma: a comparison to ganglion cell analysis and visual field.* Doc Ophthalmol, 2017. **135**(1): p. 29-42.
26. Constable, P.A., F. Marmolejo-Ramos, M. Gauthier, I.O. Lee, D.H. Skuse, and D.A. Thompson, *Discrete Wavelet Transform Analysis of the Electroretinogram in Autism Spectrum Disorder and Attention Deficit Hyperactivity Disorder.* Frontiers in Neuroscience, 2022. **16**.
27. Matsiuk, A.V. and M.V. Pryimak. *Mathematical Model of Electroretinogram in the Form of Linear Stochastic Process*. in *2005 IEEE Intelligent Data Acquisition and Advanced Computing Systems: Technology and Applications*. 2005.
28. Johnson, M.A., B.G. Jeffrey, A.M.V. Messias, and A.G. Robson, *ISCEV extended protocol for the stimulus-response series for the dark-adapted full-field ERG b-wave.* Doc Ophthalmol, 2019. **138**(3): p. 217-227.
29. Hamilton, R., M.A. Bees, C.A. Chaplin, and D.L. McCulloch, *The luminance-response function of the human photopic electroretinogram: a mathematical model.* Vision Res, 2007. **47**(23): p. 2968-72.
30. Constable, P.A., S.B. Gaigg, D.M. Bowler, H. Jagle, and D.A. Thompson, *Full-field electroretinogram in autism spectrum disorder.* Doc Ophthalmol, 2016. **132**(2): p. 83-99.
31. Lee, I.O., D.H. Skuse, P.A. Constable, F. Marmolejo-Ramos, L.R. Olsen, and D.A. Thompson, *The electroretinogram b-wave amplitude: a differential physiological measure for Attention Deficit Hyperactivity Disorder and Autism Spectrum Disorder.* J Neurodev Disord, 2022. **14**(1): p. 30.
